# Circulating miRNAs in cancer: from detection to therapy

**DOI:** 10.1186/s13045-014-0086-0

**Published:** 2014-12-05

**Authors:** Wen-Tao Wang, Yue-Qin Chen

**Affiliations:** Key Laboratory of Gene Engineering of the Ministry of Education, State Key Laboratory for Biocontrol, School of Life Science, Sun Yat-sen University, Guangzhou, 510275 P. R. China

## Abstract

Since the discovery of circulating microRNAs (miRNAs) in body fluids, an increasing number of studies have focused on their potential as non-invasive biomarkers and as therapeutic targets or tools for many diseases, particularly for cancers. Because of their stability, miRNAs are easily detectable in body fluids. Extracellular miRNAs have potential as biomarkers for the prediction and prognosis of cancer. Moreover, they also enable communication between cells within the tumor microenvironment, thereby influencing tumorigenesis. In this review, we summarize the progresses made over the past decade regarding circulating miRNAs, from the development of detection methods to their clinical application as biomarkers and therapeutic tools for cancer. We also discuss the advantages and limitations of different detection methods and the pathways of circulating miRNAs in cell-cell communication, in addition to their clinical pharmacokinetics and toxicity in human organs. Finally, we highlight the potential of circulating miRNAs in clinical applications for cancer.

## Introduction

MiRNAs are a class of small, single-stranded non-coding RNAs of 19–24 nt in length that primarily function at the posttranscriptional level [1–4]. MiRNAs function in many biological processes, such as stem cell differentiation, cell proliferation, cell apoptosis, and embryonic development [5–7]. The majority of miRNAs appear to be tissue-specific, and aberrant expression of miRNAs is associated with many diseases including cancer [8–10].

Previous studies have demonstrated that miRNAs are stable in serum and plasma and that their expression profile responds to changes under different physiological and pathological conditions [11,12]. Although circulating miRNAs may serve as biomarkers for various types of cancers [12–14], the isolation and measurement of circulating miRNAs remains a challenging task. In addition, although many studies have attempted to explain the origin and function of circulating miRNAs in cancer patients, no definitive source for these molecules has been proposed. Some cell-free miRNAs in body fluids may be packaged in exosomes, microvesicles or RNA-binding-proteins, which provide protection from RNases [15–19], and enable their transfer from one cell to another during diverse biological processes. By defining tumors as relatively homogeneous cancer cells formed in an independent microenvironment, circulating miRNAs may play a novel role as regulators of cell-cell communications during cancer formation [20,21].

In this review, we summarize recent progress made in understanding the role of circulating miRNAs in cancer, including the application of different detection methods and the quantification of serum miRNAs as non-invasive biomarkers for cancer diagnostics. We also discuss implications for serum miRNAs in cell-to-cell communication and review their roles in the development and progression of cancer and in cancer therapy.

### Detection methods to quantify serum miRNAs in cancer patients

Although a number of studies have reported that miRNAs are stable in serum and plasma and that their expression profiles change under different physiological and pathological conditions, their low enrichment in serum and the isolation and quantification of circulating miRNAs is one of the major issues in investigating circulating miRNAs. Currently, two major global miRNA profiling platforms have been suggested: microarrays and quantitative polymerase chain reaction (qPCR) based methods including relative quantification RT-PCR [14,22,23] and absolute quantitative PCR [24–26]. The methods used were shown in Table 1. The most convenient and powerful method is relative quantification RT-PCR, which has been widely used. This method can be used to detect miRNAs from either tissue samples or serum samples [22,24,27]. However, this method requires a suitable internal control, and some researchers have noted that internal controls, such as U6 [28], UNR6B [23], miR-16 [29] and miR-39 [30], are not stable or reliable. For example, circulating miR-16 can serve as an internal control in prostate cancer [29] but it is dysregulated in multiple myeloma [31] and early rheumatoid arthritis [32], thus indicating that the use of internal controls for serum requires carefully planning. To overcome this problem, some studies have suggested adding an external control to normalize the level of circulating miRNAs. The exogenous references that have been used are non-human mature miRNAs, such as cel-miR-39 [33], cel-miR-54 [26] and cel-miR-238 [34], which are spiked into the serum samples prior to RNA extraction. However, it is difficult control the amount of this artificial external control added into different serum samples. Because of the difficulties regarding endogenous controls, absolute quantitative PCR was developed. This method can be used to measure circulating miRNA expression independently, and the CT value does not require normalizing using endogenous or external controls, such as U6 or housekeeping miRNAs [35,36]. However, this method also has disadvantages; for example, the standard curve for absolute quantitative PCR for specific miRNAs must be initially constructed using a series of standards, which is labor-intensive, time-consuming and relatively costly.

**Table 1:** **Summary of the circulating miRNAs that may be used as non-invasive biomarkers for the detection of cancer**

**Circulating miRNA**	**Types of cancer**	**Methods**	**Source**	**References**
miR-155,-210,-21	Diffuse large B-cell lymphoma	qRT-PCR	serum	[14]
miR-132, -181c, -15a, -370, -143-3p, -21-5p, -200a-3p, -646	Cervical squamous cell carcinoma	TaqMan miRNA array, qRT-PCR	serum	[26]
miR-122	Chronic hepatitis B	Microarray, qRT-PCR	serum	[28]
miR-141	Prostate cancer	qRT-PCR	serum/plasma	[29,41]
miR-1233	Renal cell carcinoma	TaqMan low density array, qRT-PCR	serum	[30]
mir-16,-25	Multiple myeloma	NanoString-nCounter microRNA assays	serum	[31,52]
miR-17, -20a, -29c, -223	Nasopharyngeal carcinoma	Microarray, qRT-PCR	serum	[32]
miR-17-3p, -29a, -92a, -221	Colorectal Cancer	qRT-PCR	serum	[41,42,51]
miR-155	Breast Cancer	TaqMan miRNA array, qRT-PCR	serum	[41,49,54]
miR-21, -26a, -27a, -122, -192, -223, -801	Hepatocellular cancer	Microarray, qRT-PCR	serum	[48]
miR-16, -25, -92a, -451, miR-486-5p	Gastric non-cardia adenocarcinoma	TaqMan low density array, qRT-PCR	serum	[50]
miR-425-5p, -93-5p	Head and neck cancer	qRT-PCR	Plasma	[53]
miR-122	Chronic hepatitis C	qRT-PCR	serum	[55]

In recent years, microarray-based techniques have also been widely used to detect expression profiles of circulating miRNAs [25,26,30–32,37]. The advantage of the microarray method is its ability to assess genome-wide expression profiles of miRNAs in body fluids and to provide large amounts of candidate biomarkers for diagnostic purposes in cancer. However, this method requires a large amount of total RNA and a pre-amplification step, which may be risk of changing the original concentration of the circulating miRNAs. Chen et al. quantified circulating miRNA expression using both RT-PCR and microarray and noted a weak correlation [38], implying a risk of inaccuracy when using microarray-based methods.

In addition to the methods mentioned above, new methodologies for measuring circulating miRNAs have emerged recently. Ho and colleagues developed a self-assembling protein platform for the direct quantification of circulating miRNAs in serum based on total internal reflection fluorescence microscopy; a direct, specific and highly sensitive yet simple detection assay for miRNA that does not require sample amplification [39]. Using a self-assembled protein nanofibril and a locked nucleic acid (LNA) as the complimentary sequence and probe to capture the target miRNAs, this method can detect very low concentrations of RNA. A detection limit of 1 pM was achieved using trace amounts of sample [39]. Lusi et al. also developed a PCR- and label-free method to detect miRNA based on an electrochemical genosensor with a detection limit of up to 0.1 pmol [40,41]. Currently, some commercial PCR kits can directly detect RNA in serum or other body fluids without RNA isolation [42], which might be an important trend in circulating DNA and RNA detection, particularly for circulating miRNAs. Finally, it is worth mentioning that following the emergence of next-generation sequencing (NGS), NGS has been widely used in detecting circulating miRNAs and for the identification of novel miRNAs [43–47]. This technique does not rely on probes specific to each miRNA and is largely sequence independent, thus providing comprehensive and accurate measurements of miRNAs [43,44]; however, its disadvantages are that it is expensive and resource-consuming.

Based on the discussion above, it could be suggested that the quality of RNA is one of the most important issues for measuring circulating miRNAs. On a different note, circulating miRNAs are easily contaminated by serum cellular components, such as platelets or erythrocytes, and must be very carefully centrifuged from serum or other body fluids. This point is particularly important in the case of microarray assays. Another key issue is RNA purity and integrity, which may improve the accuracy of detection.

### Implications of circulating miRNAs as biomarkers for cancer

Circulating miRNAs have numerous advantages including stably existing in almost all body fluids, cancer-specific or tightly correlating with physiological and pathological changes and easily detectable. Consequently, a number of studies have proposed that circulating miRNAs could be ideal biomarkers for the prediction and prognosis of cancer. The first comprehensive analysis of circulating miRNAs in cancer patients was performed by Lawrie et al. They compared the expression levels of tumor-associated miR-155, miR-210 and miR-21 in serum from patients with diffuse large B-cell lymphoma with levels in serum from healthy controls and observed higher levels in patients compared with controls [14]. Mitchell et al. established an important approach when measuring tumor-derived miRNAs in serum or plasma and suggested that serum level of miR-141 can distinguish patients with prostate cancer from healthy controls [29]. In hepatocellular cancer, based on microarray and qRT-PCR, seven circulating miRNAs (miR-21, -26a, -27a, -122, -192, -223, and -801) were reported to differentiate HCC patients from healthy controls, patients with chronic hepatitis B or patients with cirrhosis [48]. Table 1 summarizes recently described circulating miRNAs that may be used as non-invasive biomarkers for cancer detection.

In further examples of the differentiation of diseased patients from healthy controls, circulating miRNAs have also been demonstrated as biomarkers for early-stage cancer diagnosis. For example, Roth et al. noted that the concentration of miR-155 in serum significantly discriminated M0- breast cancer patients from healthy women [49]. Zhu et al. also identified five miRNAs (miR-16, miR-25, miR-92a, miR-451 and miR-486-5p) as potential markers for the early-stage gastric non-cardia adenocarcinoma (GNCA) (stage I) [50]. It has been reported that circulating miR-92a was dysregulated in non-metastatic and metastatic colorectal cancer (CRC) patients and showed potential as a non-invasive biomarker for the early detection of liver metastasis in CRC patients [51].

More importantly, clinical studies have also demonstrated the potential use of miRNAs as promising biomarkers for assessing cancer prognosis. Rocci A et al. used the International Staging System (ISS) and the presence or absence of specific fluorescent in-situ hybridization abnormalities to test circulating miRNAs in patients with multiple myeloma (MM) and suggested that circulating miRNAs showed promise as new prognostic tools for multiple myeloma [52]. Pu et al. have also shown that miR-221 in the plasma could be used as a potential non-invasive prognostic biomarker for colorectal cancer [42]. Our previous study showed that eight miRNAs (miR-132, miR-181c, miR-15a, miR-370, miR-143-3p, miR-21-5p, miR-200a-3p, and miR-646) were expressed abnormally in different perioperative periods of the same cervical squamous cell carcinoma patients including pre-operative, one week post-operative and one month post-operative, suggesting that the levels of specific circulating miRNAs could be useful for post-therapeutic monitoring of the progression of this disease [22].

Recent studies revealed another important clinical application of circulating miRNAs as predictors of response to therapeutics, such as radiotherapy and anti-cancer agents. For example, Summerer et al. reported that the level of circulating miRNAs (miR-425-5p, miR-93-5p) changed dramatically in paired blood-plasma samples of head and neck squamous cell carcinoma (HNSCC) patients prior to therapy and following two days of radio chemotherapy, suggesting that they might be a novel biomarker for monitoring therapy [53]. Another study also revealed that a cluster of highly expressed miRNAs were affected by chemotherapy over the period from the initial to fourth cycle of treatment in breast-cancer patients, particularly in patients with early-stage tumors [54]. With respect to the correlation between levels of serum miR-122 and pegylated interferon therapy in patients with chronic hepatitis C, Su et al. described that patients who showed complete, early virologic response and SVR had significantly higher levels of pre-treatment serum miR-122 than those with NR, particularly sub-groups of patients with hepatitis C virus genotype 2 and IL-28B rs8099917 TT genotype. These data suggest that serum miR-122 levels may help predict virologic responses to pegylated IFN plus ribavirin therapy [55].

### Circulating miRNAs display roles in cancer development via cell-cell communication

In addition to the capacity of miRNAs as non-invasive biomarkers for cancer prediction, circulating miRNA may also play a role in the regulation of tumorigenesis. Although the underlying mechanism by which the circulating miRNAs function in the development and progression of cancer remains unclear, exosomes, which are new players in cell-cell communication and facilitate processes including antigen presentation and hemostasis, are widely researched [56–61]. A study by Parolini et al. showed that exosomes can fuse with the plasma membrane leading to the release of the exosome content into the target cell, particularly under acidic conditions.

Given that exosomes play an important role in the regulation of cell-cell communication, exosome-derived miRNA may be transferred from donor cells to receptor cells and function in the target cells in diverse biological processes. Valadi et al. were the first to report evidence of exosome-mediated delivery of RNA and circulating miRNAs from donor cells to neighboring cells [62]. Using microarray, they initially detected mRNAs and small RNAs including miRNAs in a mouse and a human mast cell line (MC/9 and HMC-1, respectively) and in primary bone marrow-derived mouse mast cells. They also reported that RNA from mast-cell exosomes was transferable to other mouse and human mast cells and that it was functional in this new location. Chen and colleagues also observed that some exosome-derived miRNAs from doc-resistant BCa cells can be robustly transferred to fluorescent sensitive cells [21]. Notably, a very recent study reported that semen can release seminal exosome (SE) preparations, which contain a large number of small RNAs, such as miRNA and tRNA [63], suggesting that SE could potentially deliver miRNAs to recipient mucosa and that may regulate embryonic development. These findings further suggest that exosomes containing miRNAs can be delivered from donor cells to recipient cells and may have the potential to be therapeutic tools to treat cancers under diverse pathological conditions.

**Figure 1:**
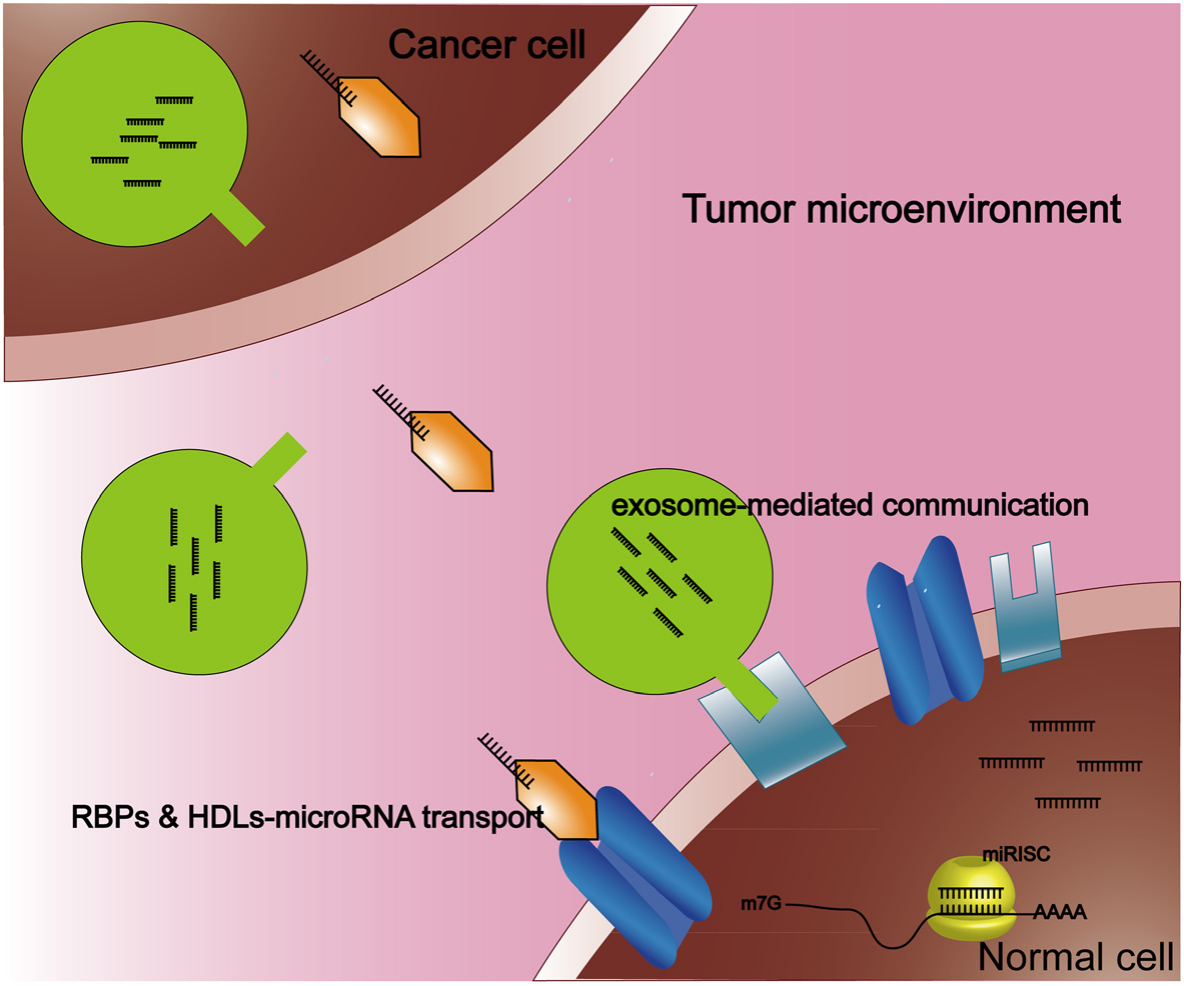
**Circulating miRNAs in cell-cell communication.** Circulating miRNAs display roles in cancer development via cell-cell communication with exosome-derived miRNAs or with miRNA-binding molecules, such as the HDL pathway, in the tumor microenvironment.

Exosome-derived miRNA delivered between cancer cells and normal cells within the tumor microenvironment also suggests that regulatory signals have the potential to play important roles in the process of tumorigenesis [58,64,65]. An example is exosome miRNAs in acute myelocytic leukemia (AML). Hornick et al. demonstrated that exosome miRNAs (miR-146, -150, 155, 210) released from circulating leukemia cells reduced hematopoiesis by modulating hematopoietic-stromal interactions, in part by targeting SDF1a, SCF, and Angpt1 during AML progression in the bone-marrow microenvironment [64]. Another report discussed exosome miR-21 in glioblastoma tumors. It showed that glioblastoma tumor cells can release exosomes containing miR-21 and angiogenic proteins, which are taken up by brain microvascular endothelial cells, and induce them to transform into cancer cells [64,66]. Yang et al. have also proposed that the exosome-derived miR-223 was released by macrophages and was transferred to co-cultivated SKBR3 and MDA-MB-231 cells, suggesting a plausible mechanism of promoting breast cancer invasion via the miR-223/Mef2c/β-catenin pathway [65]. MiR-126, shuttled by exosomes and targets tocxcl12, modulates adhesion and migration in chronic myelogenous leukemia cells [67]. These findings support the hypothesis that exosomal miRNAs have an important role in cancer-non-cancer-cell crosstalk within the tumor microenvironment and that they potentially affect disease progression (Figure 1).

In addition to exosome/nanovesicle–derived miRNAs, recent studies have also shown that circulating miRNAs can be delivered by special miRNA-binding molecules (Figure 1). For example, Vickers et al. reported that the uptake of miRNA high-density lipoprotein (HDL) in plasma by recipient cells appears to be dependent on a cell-surface HDL receptor named scavenger receptor class B, type I (SRBI) [68], suggesting a novel possible mechanism for the function of circulating miRNAs on the progression of atherosclerosis or cancer. Currently, several miRNA-binding proteins have been reported to participate in circulating miRNA inter-or extra-cellular communication, such as nucleophosmin1 (NPM1) [69] and Argonaute2 (Ago2) -1,-2 -3 and -4 [16,70–72].

Information on circulating miRNA in microvesicles or biological molecules has only appeared in recent years, and the exact role of circulating miRNAs in the development of cancer via cell-cell communication requires further investigation.

### Therapeutic applications of circulating miRNAs in cancer

**Figure 2:**
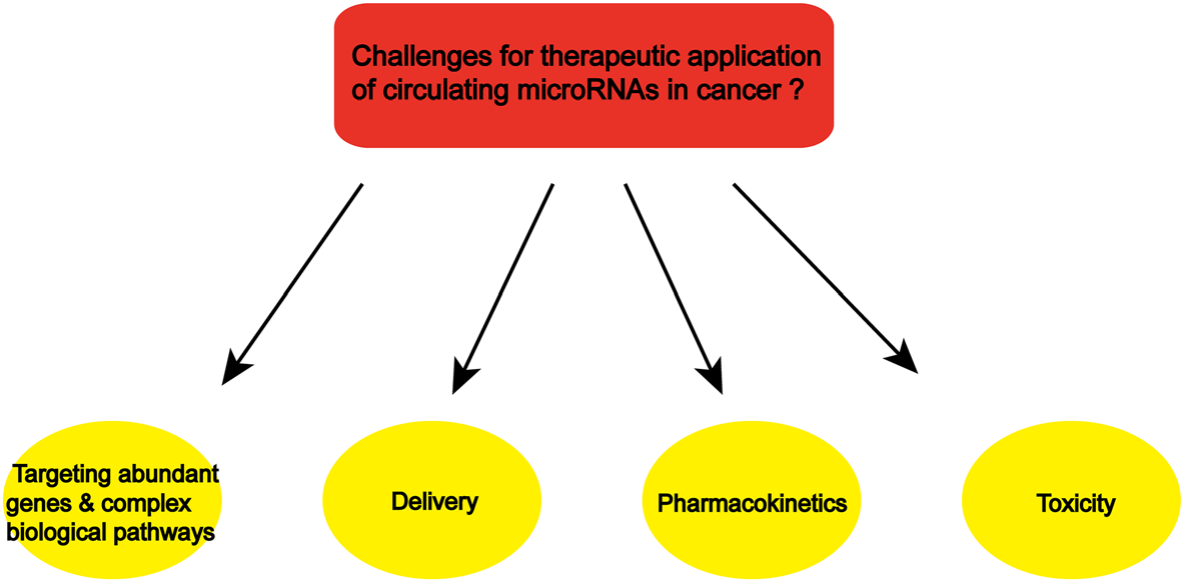
**Challenges for therapeutic implications of circulating miRNAs in cancer.**

The evidence and discussion above show that circulating miRNAs might function in the progression of tumorigenesis via cell-cell communication, thus suggesting their potential for serving as therapeutic tools in the future. More interestingly, a few studies have indicated that plant miRNAs are found in human and animal serum and that they are primarily acquired via food intake [73–76]. Exosome/microvesicle-mediated delivery of miRNAs, acquired via food intake or taken up by cells directly, could be a novel tool for cancer therapy. Although the application of miRNAs in clinical practice remains a challenge (Figure 2), there have been several reports of the development of therapeutics based on miRNA knock-down or overexpression in mice. An example is miR-135b in colon cancer. MiR-135b has been found to be up-regulated in mouse embryo fibroblasts and human colorectal cancer cell lines [77], and up-regulation of miR-135b resulted in a reduction in apoptosis and an increase in cell growth due to the down-regulation of transforming growth factor β receptor 2 (TGFβR2), death-associated protein kinase 1 (DAPK1),Adenomatous Polyposis Coli (APC), and FIH, and the activation of APC/β-catenin and SRC-PI3K pathways. This study further reported reduced proliferation and increased apoptosis in colorectal tumors in mice treated with anti-miR-135b, suggesting a preclinical efficacy of miR-135b in vivo with low toxicity. This study is the first in vivo study of anti-miRs in the treatment of colorectal cancer. Uchino et al. has also reported that the transurethral injection of synthetic miR-582 suppressed tumor growth and metastasis in a mouse model of bladder cancer [78].

However, several problems have been encountered during clinical development (Figure 2) of exosome miRNA-based therapeutics for tumors. One of the major challenges is that miRNAs target numerous genes in diverse types of cells, and their functions in the complex networks of biological pathways remain unclear. For example, miR-125 plays the role of angel and devil in different cancer types [79]. With respect to ovarian cancer [80], bladder cancer [81], breast cancer [82] and some other cancers, miR-125 suppresses tumors by targeting oncogenes, whereas in other types of cancers, such as pancreatic cancer [83], prostate cancer [84] and oligodendroglial cancer [85], miR-125 itself acts as an oncogene. The fact that one miRNA has varying functions makes miRNA-based therapeutics difficult. Another major issue is the difficulties in obtaining accurate quantification of exosome-derived miRNAs due to different measurement methodologies or degradation in different ways [86], and the consequent variation might influence therapeutic effects.

Furthermore, the delivery of circulating miRNA antagonists or mimics as cancer therapies also encounters several barriers, such as poor bioavailability, limited tissue permeability and payload stability [87]. For example, the fibrotic microenvironment of pancreatic cancer results in poor diffusion of therapeutic drugs and suggests that the complex tumor microenvironment and extracellular matrix (ECM) may hinder miRNAs from reaching the cancer cells [88]. Castelliet et al. also showed that the tumor-associated macrophages can non-specifically take up and trap miRNAs encapsulated in a delivery system [89], which may lead to poor bioavailability of miRNAs. The local delivery of miRNA by intra-tumoral injection may improve delivery and enable effective gene-silencing and anti-tumor effects [87]. Some reports have shown the successful delivery of siRNA via electroporation or spherical nucleic acid nanoparticle conjugates (SNA-NCs), resulting in direct silencing of the target gene in skin [90,91]. However, the local delivery of miRNA may enhance therapeutic effects only in the primary tumor that resides in specific locations and may not be suitable for late-stage metastatic cancer. Thus, a method of systemic delivery is required for cancer therapy.

Finally, the most important issue for miRNAs is clinical pharmacokinetics and toxicity of miRNAs in human organs. Recent studies have demonstrated that miRNAs are highly water soluble and stable in serum. However, the plasma levels of miRNAs declines rapidly following food intake or intravenous administration, and they spread widely through the internal environment but may later accumulate primarily in the liver and kidney [92]. Thus, tracing and measuring circulating miRNAs in target tumor cells and monitoring pharmacological effects are important and challenging issues. Induction of toxicity due to off-target effects of miRNAs or a systemic immune response to miRNA injection may influence the progression of clinical applications. For example, following liposome transfection of synthetic miR-145 into human mesenchymal stem cells and human articular chondrocytes, Tommy and Jan observed immunological off-target effects that were independent of toll-like receptors (TLRs) and that were mediated by retinoic acid inducible-gene 1 (RIG-I) [93]. To overcome this immunotoxicity, miRNAs may be delivered by exosomes or vesicles incorporated with specific ligands or antibodies or that bind endogenous receptors of cancer cells. Neesse et al. constructed albumin-conjugated nanoparticles enriched with cysteine (SPARC), which is robustly expressed by pancreatic adenocarcinoma [94], and efficiently delivered the drug to the tumor [95]. Thus, the specific exosomes containing miRNAs may provide an alternative to enable cancer treatment without toxicity, resulting in accurate uptake of therapeutic miRNAs in the tumor site and suppressed significantly the tumor regression.

## Conclusions and perspectives

In this review, we summarized recent proresses on circulating miRNAs from the development of detection methods and their clinical applications as biomarkers to their role as regulators of tumorigenesis and as cancer therapeutics. Progresses on the biology of miRNAs greatly enhance our understanding of their functions with respect to basic mechanisms of oncogenesis and diagnostics and prognostics. Clearly, cell-free miRNAs might play an increasing role as non-invasive tools for the detection of cancer in early stages and as biomarker to monitor prognosis and response to therapy. However, knowledge on the biological functions of extracellular miRNAs is in its infancy. Recent studies revealed that circulating miRNAs are derived from different sources, that they may represent varying human physiological states and that they may play roles in the development of cancer via cell-cell communication. With the finding that exosome miRNA transfers are dependent on cell-cell communication, circulating miRNAs demonstrate their potential for therapeutic application in cancer.

Although great progress in the detection and function of circulating miRNAs in cancer has been made, some challenges exist in their clinical application and require further investigation. The application of direct amplification of circulating miRNAs from plasma without RNA extraction will be a novel approach that shortens the procedure and provides more realistic and original data for analysis. With the development of more effective and specific PCR enzymes for miRNA quantification, the detection of circulating miRNAs for direct cancer prediction in the clinic will be possible in the future.

Another major challenge is to demonstrate the exact mechanism of circulating miRNAs derived from dead or lysing cells or secreted from tumor cells and the mechanisms of their movement, signal transmission or their role in oncogenesis. Although recent studies have suggested that circulating miRNA appears to be transported from cell to cell via the extracellular matrix either by a ligand-receptor system or via their ability to diffuse into adjacent cells, or endosomal vesicles can move along microtubulin to locate to another organelle [96–99], the exact mechanisms deserve further investigation.

In addition to the potential of circulating miRNAs as biomarkers for cancer prediction or prognosis, circulating microRNAs may enable personalized cancer medicine in future. However, several issues must be solved before going further. One of these issues is the limited tissue permeability of miRNAs and payload stability. The commercial miRNA mimics or antagonists are unstable and degrade easily when loaded into exosomes. Modification of miRNAs may increase their stability and avoid RNA enzyme activity when delivered in vivo. Thus, research to improve the stability of exosome loading of miRNAs without reducing pharmacological effects is urgent. In addition, to overcome poor cancer-tissue permeability, some exosome/vesicles incorporated with ligands or antibodies or nanoparticles containing miRNAs may be designed. These modified complexes may not only enhance the permeability of the tumor but also decrease the immunotoxicity of the complex and decrease its induction of the human immune system. Cell-based delivery of miRNA mimics or antagonists may also enable the development of cures for early-stage cancer [87]. Furthermore, the pharmacokinetics of circulating miRNAs is also a challenge in the clinic when administering them via food intake. Preclinical studies in animals may aid with these investigations over the coming years. Finally, although there are large numbers of reports on the functions of miRNAs in the development of cancer, the complex gene networks affected by them remains a considerable problem. The complex relationships between miRNAs and their functional pathways must be demonstrated.

With the understanding of the origin, stability, their role in cell-cell communication and advances in methods to detect circulating miRNAs, we believe that, in the future, they will become a cancer buster: From detection to therapy.
